# Can a single question replace patient-reported outcomes in the follow-up of elbow arthroplasty? A validation study

**DOI:** 10.1186/s10195-024-00790-2

**Published:** 2024-10-22

**Authors:** Arno A. Macken, Ante Prkic, Iris Koenraadt-van Oost, Geert A. Buijze, Bertram The, Denise Eygendaal

**Affiliations:** 1 Department of Orthopaedics and Sports Medicine, Erasmus Medical Centre, Dr Molenwaterplein 40, 3015 GD Rotterdam, The Netherlands; 2 Alps Surgery Institute, Clinique Générale Annecy, 4 Chemin de La Tour La Reine, 74000 Annecy, France; 3 Department of Orthopaedic Surgery, Amphia Hospital, Molengracht 21, 4818 CK Breda, The Netherlands; 4 Department of Orthopaedic Surgery, Amsterdam UMC, Meibergdreef 9, 1105 AZ Amsterdam, The Netherlands; 5 Department of Orthopedic Surgery, Montpellier University Medical Center, 291 Avenue du Doyen Gaston Giraud, 34000 Montpellier, France

**Keywords:** Elbow arthroplasty, Single assessment numeric evaluation, Subjective elbow score, Patient-reported outcomes, Follow-up

## Abstract

**Background:**

To assess the results after elbow arthroplasty it is essential to gather patient-reported outcome measures (PROMs). However, the acquisition of PROMs poses a challenge because of potential low literacy, lengthiness and diversity of questionnaires, and questionnaire fatigue. Instead of a questionnaire, patient-reported outcomes can be collected using a single assessment numeric evaluation (SANE), the subjective elbow value (SEV). The aim of this pilot study is to assess the correlation between the SEV and conventionally used patient reported outcome measures (PROMs) after elbow arthroplasty.

**Materials and methods:**

The SEV was added to our follow-up system in 2021, consisting of a scale from 0 to 10 in which the patients are asked to rate the overall functionality of their elbow, 0 corresponds to very poor functionality and 10 to a perfectly functional or healthy elbow. All patients who underwent elbow arthroplasty (total or radial head) and responded to the SEV question were retrospectively identified and included. The correlation between the SEV at the final follow-up and the Oxford Elbow Score (OES), and between the SEV and the Quick Disbailities of the Arm, Shoulder, and Hand (quickDASH) score was assessed using Pearson’s *r*.

**Results:**

In total, 82 patients responded to the SEV question and were included in the study, with a median follow-up of 5 years [interquartile range (IQR) 3–7]. Of these patients, 17 (21%) underwent radial head arthroplasty and 65 (79%) total elbow arthroplasty. The Pearson’s *r* for the correlation between SEV and OES was 0.502 (*p* < 0.001) and between the SEV and the QuickDASH −0.537 (*p* < 0.001), which correspond to a moderate correlation.

**Conclusions:**

The SEV shows a moderate correlation with conventional PROMs, demonstrating its potential in simplifying the follow-up of elbow arthroplasty, possibly decreasing time, costs, and patients’ questionnaire fatigue compared with conventional PROM questionnaires.

*Evidence level*: III.

## Introduction

To assess the results of an intervention it is essential to gather patient-reported outcome measures (PROMs). Interest in PROMs in orthopedics has increased dramatically over the last few decades, as is demonstrated by a comparison of PubMed (MEDLINE) search terms for PROMs yielding 500 results in 2000 and 6188 results in 2020. However, the acquisition of PROMs poses a challenge because of potential low literacy, lengthiness and diversity of questionnaires, time burden, questionnaire fatigue, and data collection issues. Many different PROMs are used, complicating the comparison of results. There is currently no consensus on which outcomes should be gathered after elbow arthroplasty.

Instead of a questionnaire, patient-reported outcomes can be collected using a single question assessing the overall functionality of the joint, as is demonstrated by the Subjective Shoulder Value (SSV) [9]. A single assessment numeric evaluation (SANE), such as the SSV is commonly used in shoulder research, with some authors arguing that it can be used as a stand-alone outcome instrument after shoulder procedures [2]. Previous studies report moderate to high correlation of the SSV with conventional shoulder PROMs (*r* = 0.50–0.88) [15]. Furthermore, the test–retest reliability of the SSV has been shown to be similar to that of the American Shoulder and Elbow Surgeons (ASES) score [Interclass correlation (ICC) = 0.84 versus ICC = 0.82], suggesting that the SSV is at least as reliable as more complex PROMs [23]. However, relatively very few studies report a SANE as a follow-up metric after elbow procedures or injuries, such as the Subjective Elbow Value (SEV) [1, 3, 7, 8, 13, 14, 16, 19].

In previous literature, moderate to high correlations were found between the SANE for the elbow and conventional elbow PROMs administered during outpatient clinic visits for elbow-related problems, such as the Oxford Elbow Score (OES; *r* = 0.903) and the ASES-Elbow (*r* = 0.623), and the physician-administered Mayo Elbow Performance Index (MEPI; *r* = 0.671) [17, 18, 20, 21]. However, for more specific PROMs, such as the Patient-rated Tennis Elbow Evaluation (PRTEE), a weaker correlation was found (*r* = 0.391), although statistically significant (*p* = 0.017) [10]. The elbow conditions in these studies are very heterogeneous and mostly concern first-time clinical visits, they do not assess the SEV as outcome metric during follow-up for specific procedures. Furthermore, many studies reporting the SEV concern sports injuries [6, 13, 14, 16].

Three studies attempted to validate the SEV as an outcome metric after elbow injuries reporting high correlations with Disability of the Arm, Shoulder, and Hand (DASH; *r* = − 0.85), MEPI (*r* = 0.80 and *r* = 0.710, respectively), and OES (*r* = 0.764 and *r* = 0.76) [6, 8, 12]. However, the outcomes were concentrated toward the positive end of the scale in two out of the three studies (mean SEV 90% and 87%), which increases homogeneity. For other procedures, such as arthroplasty, no previous studies assess the correlation of SEV with conventional PROMs as an outcome metric. However, simplification of the follow-up after arthroplasty is especially relevant. Besides decreasing patient burden and costs, it may also be used for arthroplasty registries to increase simplicity and uniformity, thereby facilitating international comparison and collaboration between arthroplasty registries.

The aim of this pilot study is to establish whether a Single Assessment Numeric Evaluation (SANE), the Subjective Elbow Value (SEV), is correlated to conventionally used Patient Reported Outcome Measures (PROMs) after elbow arthroplasty, which could lead to a simplification and reduction of questionnaires during follow-up. Moreover, it could serve as a simple, uniform question for (inter)national elbow arthroplasty registries, which is currently lacking.

## Materials and methods

The protocol for this study was reviewed and approved by the institutional review board. Patients that undergo an elbow prosthesis at our institution are routinely contacted by email for follow-up 1, 3, 5, 7, and 10 years after surgery, and every 5 years thereafter. A Single Assessment Numeric Evaluation (SANE) question, the Subjective Elbow Value (SEV), was added to the follow-up system in 2021. This question consists of a scale from 0 to 10 in which the patients are asked to rate the overall functionality of their elbow, 0 corresponds to very poor functionality and 10 to a perfectly functional or healthy elbow. The SEV can also be expressed in percentages (0–100%). All patients that underwent primary total elbow arthroplasty (TEA) or radial head arthroplasty (RHA) in our center’s online follow-up system (onlinePROMS, 's-Hertogenbosch, the Netherlands), operated between January 2012 and June 2022, were retrospectively identified. Inclusion criteria were: patients that underwent primary total elbow or radial head arthroplasty for any indication, a minimum follow-up of 1 year, and at least one response to the SEV question in the follow-up system. Patients without a response to the SEV question in the follow-up system were excluded. Patient and treatment characteristics were extracted from the local registry and patients’ charts.

Statistical analysis was performed according to a predefined plan. Descriptive statistics, including means or medians and standard deviations (SD) or interquartile ranges (IQR), were calculated for the demographic and surgical data. For the primary hypothesis of this study, the size and significance of the correlation between the SEV and the OES, and between the SEV and the quickDASH score was assessed. Only the most recent follow-up including the SEV question was used for the analysis, regardless of whether patients had undergone revision surgery. All other outcomes from the same follow-up period were used. The correlation between the SEV and the PROMs was visualized using scatterplots. In case of a linear correlation without significant outliers, Pearson’s *r* was used to assess the strength of the correlation between the two variables. Pearson’s test results in a *p*-value representing the statistical significance of the correlation and a correlation size ranging from −1 (strong negative correlation) to 0 (no correlation) and 1 (strong positive correlation). A value from 0.9–1.0 is considered a very high correlation, 0.7–0.9 high correlation, 0.5–0.7 moderate correlation, 0.3–0.5 low correlation, and 0–0.3 negligible correlation. The same classification applies to negative numbers for negative correlations. In case of a nonlinear correlation, transformations were attempted to arrive at a linear correlation. In case no transformation leads to a linear correlation, Spearman’s ρ was used. In case of significant outliers Kendall’s τ was used.

In addition, linear regression models were built with OES and quickDASH as the outcome and the SEV as the independent variable together with the two other single numeric questions (visual analogue scale; VAS for pain in rest and VAS for pain during activity). Next, independent variables without a significant correlation to the outcome were removed. The initial and final regression models were reported. For the final model, multicollinearity was assessed using variance inflation factors. A variance inflation factor below 2.5 was considered acceptable. Furthermore, normality of the residuals was tested using QQ plots.

A *p*-value of 0.05 was considered statistically significant. Statistical analysis was performed using R version 4.0.5 (R Foundation for Statistical Computing, Vienna, Austria).

## Results

### Cohort

After approval of the institutional review board, 296 patients were retrospectively identified in the online follow-up system. Overall, 214 patients did not respond to the follow-up request or did not reach one of the standardized follow-up time-points since the introduction of the SEV in 2021 and were excluded. In total, 82 patients responded to the SEV question and were included in the study, with a median follow-up of 5 years (IQR 3–7). Of these patients, 17 (21%) underwent RHA and 65 (79%) TEA.

The mean age was 67 years (SD 9) and the majority of patients were female (77%; Table 1). The most common indication for surgery was posttraumatic sequelae (43%), followed by an acute fracture (18%), and rheumatoid arthritis (18%; Table 2). Preoperatively, patients reported a median VAS pain score of 8.4 (IQR 7.1–9.3) during activity and 5.2 (IQR 3.0–6.9) in rest. The mean preoperative OES was 16 (SD 8).

**Table 1:** Cohort characteristics (*n* = 82)

Total elbow arthroplasty, *n* (%)	65 (79)
Radial head arthroplasty, *n* (%)	17 (21)
Female, *n* (%)	66 (77)
Age, mean years (SD)	67 (9)
Smoking, *n* (%)	5 (6)
BMI, mean kg/m^2^ (SD)	27 (5)
ASA classification, *n* (%)	
I	7 (8)
II	53 (82)
III	15 (82)
IV	1 (1)
Previous surgery, *n* (%)	39 (48)
Arthroscopy	8 (10)
Arthrotomy	22 (27)
Osteosynthesis	18 (22)
Material removal	6 (7)

*ASA* American Society of Anesthesiologists, *BMI* body mass index, *SD* standard deviation

**Table 2:** Treatment characteristics (*n* = 82)

Surgical indication, *n* (%)	
Posttraumatic	36 (43)
Acute fracture	15 (18)
Rheumatoid arthritis	15 (18)
Osteoarthritis	10 (12)
Revision to TEA	4 (5)
Other or unknown	3 (3)
Left side affected, *n* (%)	48 (59)
Surgical approach, *n* (%)	
Posterior, triceps-on	39 (48)
Posterior, triceps-flap	25 (30)
Lateral, LCL intact	10 (12)
Lateral, LCL detached	4 (5)
Other or unknown	5 (6)
Cemented, *n* (%)	74 (90)
Bone graft, *n* (%)	5 (6)
Follow-up, median years (IQR)	5 (3–7)

*IQR* interquartile range, *SD* standard deviation, *TEA* total elbow arthroplasty

Two fellowship-trained, specialized elbow surgeons performed the procedures on all patients in the cohort. The left side was operated in 48 patients (59%) and the right side in 34 (41%), there were no cases of bilateral arthroplasty. For the TEAs, a Coonrad–Morrey total elbow prosthesis (ZimmerBiomet, Warsaw, Indiana, United States) was used in 80 cases (98%) and a Latitude (Wright Medical Group, Memphis, Tennessee, United States) in two cases (2%). For all RHA cases, a Tornier Radial Head System was used (Wright Medical Group).

In total, 18 patients (22%) underwent a secondary intervention to the ipsilateral elbow at a median of 8.5 months after the primary procedure (IQR 2.3–29). There were six cases of infection (five deep, one superficial), four cases in which the ulnar nerve was released, and two cases in which a contracture was released. Furthermore, there were single cases of proximal radio-ulnar osteoarthritis, aseptic loosening, bushing wear, link breakage, periprosthetic fracture, and wound necrosis. Two cases required a third intervention for a contracture and aseptic loosening of the ulnar component.

### Primary outcomes

At the final follow-up, the median SEV in the cohort was 8 (IQR 7–8). The median VAS for pain during rest was 0.8 (IQR 0.2–2.4) and the median VAS for pain during activity was 1.7 (IQR 0.5–4.8). The median OES was 36 (IQR 29–41) and the median QuickDASH score was 25 (IQR 18–52).

**Fig. 1:**
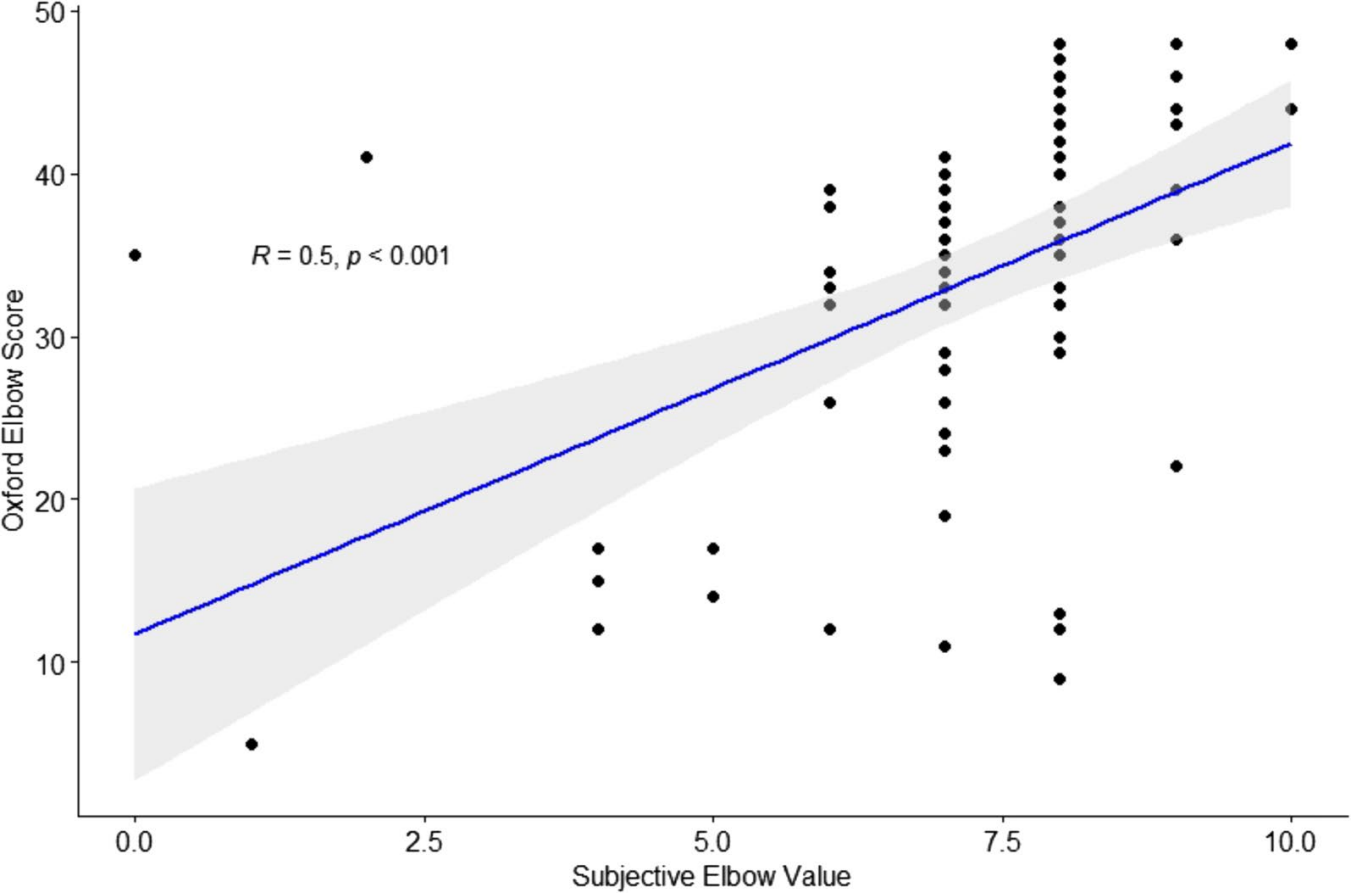
Scatterplot showing the correlation between the Subjective Elbow Value and Oxford Elbow Score, showing a statistically significant correlation (*p* < 0.001) with a Pearson’s *r* of 0.5 (moderate correlation)

Correlation of the SEV with the OES resulted in a Pearson’s *r* of 0.502 (*p* < 0.001), which corresponds to a statistically significant, moderate correlation (Fig. 1).

**Fig. 2:**
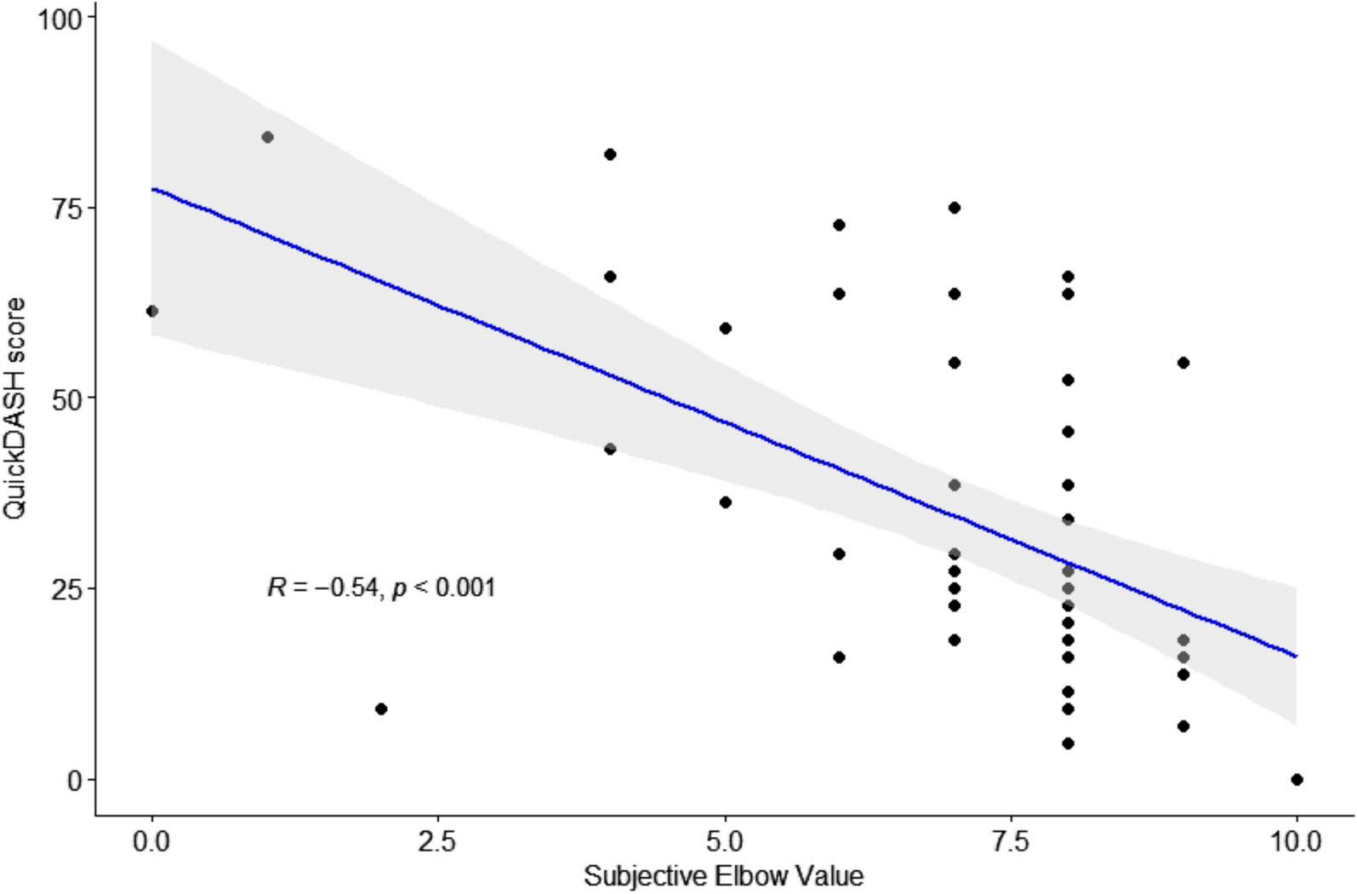
Scatterplot showing the correlation between the Subjective Elbow Value and Quick Disabilities of the Arm, Shoulder, and Hand score, showing a statistically significant correlation (*p* < 0.001) with a Pearson’s *r* of −0.54 (moderate correlation)

Correlation of the SEV with the QuickDASH score resulted in a Pearson’s *r* of −0.537 (*p* < 0.001), which corresponds to a statistically significant, moderate correlation (Fig. 2).

### Regression analyses

In the regression analyses, the SEV and VAS for pain during activity were significantly associated with OES and QuickDASH, (Tables 3 and 4) while the VAS for pain in rest was not significant (*p* = 0.636 and *p* = 0.771, respectively). In the final models the adjusted *R*^2^ was 0.5056 and 0.6302, meaning that the variables in the model explain 51 and 63% of the variance in the outcome. For both outcomes (OES and QuickDASH) the *R*^2^ improved when the VAS for pain during activity was added (0.240 to 0.506 and 0.275 to 0.630, respectively).

**Table 3:** Linear regression for OES

	Coefficient	Error	*t*-value	*p*-value	VIF
Initial model					
SANE	3.013	0.600	5.030	< 0.00001	
Adjusted *R*^2^: 0.2399					
Final model					
SANE	1.602	0.539	2.975	0.00396	1.223
VAS activity	−0.212	0.034	−6.345	< 0.00001	1.223
Adjusted *R*^2^: 0.5056,					

*SANE* single assessment numeric evaluation, *VAS* visual analogue scale, *VIF* variance inflation factor

**Table 4:** Linear regression for QuickDASH

	Coefficient	Error	*t*-value	*p*-value	VIF
Initial model					
SANE	−6.146	1.302	−4.720	< 0.00001	
Adjusted *R*^2^: 0.2754					
Final model					
SANE	−2.898	1.030	−2.813	0.00683	1.223
VAS activity	0.517	0.070	7.332	< 0.00001	1.223
Adjusted *R*^2^: 0.6302					

*SANE* Single assessment numeric evaluation, *VAS* visual analogue scale, *VIF* variance inflation factor

## Discussion

This study aimed to assess the correlation between a SANE for the elbow, the SEV, and commonly used PROMs, the OES and QuickDASH score, for the follow-up of RHA and TEA. Moderate but statistically significant correlations were found for both PROMs (*r* = 0.50 and *r* = −0.54). Furthermore, the regression analyses showed that a VAS for pain during activity is of added value to the SEV.

### Correlation with conventional PROMs

Despite the common use of a SANE, the SSV, in shoulder research [2, 15, 23], relatively few studies have attempted to validate the SEV for elbow pathology. Some studies have correlated the SEV with other patient-reported metrics in patients during a first-time visit to the outpatient clinic. In previous studies the SANE for the elbow was proven to be highly correlated to the OES (*r* = 0.903), and moderately correlated to the ASES-E (*r* = 0.623) and the physician-administered MEPI (*r* = 0.671) during outpatient clinic visits for elbow-related problems [17, 18, 20, 21]. However, for more specific metrics, the SEV showed a lower correlation. For example, in one study, a low correlation was found between the SEV and the PRTEE (*r* = 0.391) scale [10]. In all studies, the correlation was significant. The correlations found in the current study when using the SEV for follow-up (*r* = 0.50 and *r* = − 0.54) is generally lower than those found in patients presenting with new elbow pathology.

Few studies have assessed the SEV as a follow-up metric after elbow procedures or injuries. In one study of 40 patients that underwent fixation of an olecranon fracture, the SEV was highly correlated with the DASH score (**r** = − 0.85) and MEPI (*r* = 0.80) [8]. In another study of 114 patients following an elbow dislocation, the SEV was also highly correlated with both MEPI (*r* = 0.710) and OES (*r* = 0.764) [6]. One study of 75 patients with varying elbow pathology assessing the correlation between SEV and OES before injury, 1 week after injury, and 3–5 months after injury found an equally high correlation (*r* = 0.76) [12]. These results are markedly higher than the correlations with OES and QuickDASH found in the current study (*r* = 0.50 and *r* = −0.54). Several factors may explain this discrepancy. First, the MEPI score is a partly physician-assessed and partly patient-reported, potentially leading to differences in the results. In the current study, only correlations with patient-reported outcomes were assessed. Furthermore, the SEV in both studies was high (median 90% and mean 87%, respectively). This suggest that outcomes were concentrated toward the positive part of the spectrum, thereby increasing heterogeneity and leading to a higher correlation. In the current study, the median SEV was 80%. The discrepancy may also be related to the nature of the procedure or condition, it is possible that follow-up of elbow arthroplasty requires a more multifactorial approach than less complicated procedures or less severe conditions.

Interestingly, in the current study, there were several outliers of patients who reported a high OES or low DASH score in combination with a low SEV. It is possible that this is related to a misinterpretation of the SANE question, reversing the score. However, the question includes examples (0 = very poor functionality and 10 = a perfect elbow) and is not easily misinterpreted. It is also possible that low literacy in certain patients leads to a misinterpretation of the OES or quickDASH. Another explanation could be that some patients have higher demands of their elbow in daily activities or higher preoperative expectations, resulting in a mismatch between objective functionality and the SEV. Furthermore, a previous study in shoulder arthroplasty showed that preoperative Mental Component Score was correlated with postoperative pain and functional scores, suggesting that psychological aspects may also play a role in the perception of post-operative upper extremity function [4].

### Can the SEV be a standalone metric?

In the current study, the correlation between the SEV and conventional PROMs was moderate, suggesting that it is insufficient to replace these methods as a standalone follow-up metric for elbow arthroplasty. However, the true value of a follow-up metric should not be compared with the current golden standard but to the goal of the metric and the added value to the patient and healthcare provider. Common reasons to use validated follow-up metrics are to assess and report results, to detect a deterioration in the results, or for early prediction of failure of the intervention. Unfortunately, these goals could not be directly assessed in the current study. It is possible that for these purposes, the SEV performs equally well or even superior to the current standard PROMs. Furthermore, since the SEV directly asks the patient what they think of their elbow function, the result will also more closely reflect patient satisfaction. Future studies may focus on clarifying the usefulness of the SEV, not just by correlating it with existing PROMs, but by assessing its value in attaining the abovementioned goals.

The regression analyses in the current study showed that a single question VAS score for pain during activity was of added value for predicting the variation in both OES and QuickDASH scores. This is demonstrated by an increase in *R*^2^ when adding the VAS score to the model, meaning that the ability of the model to predict the variance in the outcome is increased. The VAS score for pain during rest was not of added value. This suggest that the SEV is not sufficient as a standalone metric for the follow-up of elbow arthroplasty. However, adding one or more “single-question” metrics may be sufficient for obtaining the same results as several longer questionnaires. Although not assessed in the current study, a question assessing psychological factors may also be of added value. Previous studies have shown that many psychological factors, such as depression, resilience, pain catastrophizing, and kinesiophobia, may influence PROMs in orthopedic conditions [4, 5, 11, 22]. Future studies may identify which specific SANE should belong to the core set of questions used for the follow-up of elbow conditions, potentially replacing several longer questionnaires by a handful of focused questions.

### Limitations

The results of this study must be interpreted in light of its limitations. First, the SEV was only recently introduced in our center’s follow-up system. Therefore, a substantial number of cases was excluded due to the patients not having reached their follow-up timepoint since the introduction of the SEV. Second, owing to the relatively small cohort and differences in follow-up, there was not enough follow-up data to assess each timepoint separately or to assess the evolution of the SEV over time. Instead, we opted to use the most recent follow-up for each patient. Furthermore, preoperative SEV was not recorded in most patients. Therefore, improvement from preoperative measurements to the final follow-up could not be assessed. In addition, the cohort was too small to statistically correct for confounding factors, such as the use of pain medication, which may influence PROMs. Last, only the correlation between the SEV and conventional PROMs was assessed in this study. Other validation requirements, such as the test-retest reliability and responsiveness, could not be assessed.

## Conclusions

A statistically significant, but moderate correlation was found between the SEV and the OES and QuickDASH scores as a follow-up metric after elbow arthroplasty. Although the SEV may not be sufficient as a standalone metric, it shows potential in simplifying the follow-up of elbow arthroplasty, possibly decreasing time, costs, and patients’ questionnaire fatigue compared with conventional PROM questionnaires. Future studies may identify a core set of “single-question” assessments that may be used for the follow-up of elbow arthroplasty and attempt to simplify and replace conventional PROMs.

## Data Availability

The datasets used and/or analyzed during the current study are available from the corresponding author upon reasonable request.

## Abbreviations

ASES: American Shoulder and Elbow Surgeons
DASH: Disabilities of the Arm, Shoulder, and Hand
ICC: Interclass correlation
IQR: Interquartile range
MEPI: Mayo Elbow Performance Index
OES: Oxford Elbow Score
PROM: Patient-reported outcome measure
PRTEE: Patient-rated tennis elbow evaluation
RHA: Radial head arthroplasty
SANE: Single assessment numeric evaluation
SD: Standard deviation
SEV: Subjective elbow value
SSV: Subjective shoulder value
TEA: Total elbow arthroplasty
